# The genomes of closely related *Pantoea ananatis* maize seed endophytes having different effects on the host plant differ in secretion system genes and mobile genetic elements

**DOI:** 10.3389/fmicb.2015.00440

**Published:** 2015-05-12

**Authors:** Raheleh Sheibani-Tezerji, Muhammad Naveed, Marc-André Jehl, Angela Sessitsch, Thomas Rattei, Birgit Mitter

**Affiliations:** ^1^Bioresources Unit, Health and Environment Department, AIT Austrian Institute of Technology GmbHTulln, Austria; ^2^Division of Computational System Biology, Department of Microbiology and Ecosystem Science, University of ViennaVienna, Austria

**Keywords:** seed endophyte, *Pantoea ananatis*, comparative genomics, plant growth promotion

## Abstract

The seed as a habitat for microorganisms is as yet under-explored and has quite distinct characteristics as compared to other vegetative plant tissues. In this study, we investigated three closely related *P. ananatis* strains (named S6, S7, and S8), which were isolated from maize seeds of healthy plants. Plant inoculation experiments revealed that each of these strains exhibited a different phenotype ranging from weak pathogenic (S7), commensal (S8), to a beneficial, growth-promoting effect (S6) in maize. We performed a comparative genomics analysis in order to find genetic determinants responsible for the differences observed. Recent studies provided exciting insight into the genetic drivers of niche adaption and functional diversification of the genus *Pantoea*. However, we report here for the first time on the analysis of *P. ananatis* strains colonizing the same ecological niche but showing distinct interaction strategies with the host plant. Our comparative analysis revealed that genomes of these three strains are highly similar. However, genomic differences in genes encoding protein secretion systems and putative effectors, and transposase/integrases/phage related genes could be observed.

## Introduction

Bacterial endophytes have been defined as “bacteria, which for all or part of their life cycle invade the tissues of living plants and cause unapparent and asymptomatic infections entirely within plant tissues, but cause no symptoms of disease” (Wilson, 1995). Based on this definition, endophytes are clearly distinct from plant pathogens. However, bacteria can exist in plants in quiescence but proliferate and become detrimental to the host under certain conditions such as plant growth perturbations (Kloepper et al., 2013). Moreover, plant-pathogen interactions are often plant species specific and bacteria that are pathogenic to one plant species can exhibit an endophytic lifestyle in other plants (Bashan et al., 1982). On the other hand, it has been shown that plants respond differently to endophytes and plant pathogens (Bordiec et al., 2011). A promising approach in revealing differences in the host interaction strategies of pathogens and plant beneficial bacteria might be the comparison of functionalities and gene content of closely related bacterial strains that show different modes of interaction with host plants. Genome sequencing provides detailed information on the genes present in bacteria and offers a basis for comparative genomics that aids in revealing differences in the host interaction strategies of pathogens and plant growth promoting bacteria.

The seed as a habitat for microorganisms is under-explored, although the first report of bacteria colonizing seeds dates back to the 1970s (Mundt and Hinkle, 1976). Only few studies have been performed on seed endophytes (Compant et al., 2011; Johnston-Monje and Raizada, 2011; Hardoim et al., 2012) and the origin of endophytes is under debate. A few studies suggest that at least some bacterial endophytes are vertically transmitted (Johnston-Monje and Raizada, 2011). The seed has quite distinct characteristics as compared to other vegetative plant tissues and one would expect that it also harbors distinct microbial communities. Based on cultivation-based analysis it has been reported that Gammaproteobacteria represent the most abundant class of maize seed endophytes, comprising mostly *Pantoea* and *Enterobacter* (Johnston-Monje and Raizada, 2011). Similarly, Rijavec et al. (2007) identified *Pantoea* as a major genus among endophytes isolated from maize seeds.

*Pantoea ananatis* is a bacterial species that was originally discovered in pineapple in the Philippines, in 1928 (Serrano, 1928). Members of this species have been shown to infect many mono- and dicotyledonous plant species, such as onion, rice, melon, sudan grass, tomato, and sorghum (Stall et al., 1969; Wells et al., 1987; Gitaitis and Gay, 1997; Azad et al., 2000; Cother et al., 2004; Cota et al., 2010). In maize *P. ananatis* is the causing agent of the foliar disease termed maize white spot disease (Paccola-Meirelles et al., 2001). *P. ananatis* strains display a wide range of ecological versatility, as they are commonly recovered from water, soil, insects, and plants (De Maayer et al., 2014). Depending on their host and ecological niches, *P. ananatis* strains can show different life styles such as mutualistic, saprophytic and pathogenic life styles (Coutinho and Venter, 2009). De Maayer et al. (2012a) showed that the Large Pantoea Plasmid (LLP-1) plays a crucial role in niche adaption and functional diversification of the genus *Pantoea*. By analyzing the pan-genome of eight sequenced *P. ananatis* strains De Maayer et al. (2014) identified a large number of proteins in this species with orthologs restricted to bacteria associated either with plants, animals or insects. The mechanisms of the diverse interactions between *P. ananatis* and the host are still poorly understood and only little is known on the genetic traits underlying plant pathogenic or beneficial activity. Shyntum et al. (2014) showed that type IV section system could play a role in pathogenicity and niche adaptation. Genome analysis of the plant growth promoting strain *P. ananatis* B1-9 that has been isolated from the rhizosphere of green onions in Korea indicates that the strain lacks traits related to pathogenicity. Furthermore, it harbors genes that are putatively involved in plant growth stimulation and yield improvement (Kim et al., 2012).

In this work, we studied three endophytic *P*. *ananatis* strains (S6, S7, S8) isolated from maize seeds. Although they were isolated from seeds of healthy plants, they showed distinct characteristics in regard to plant growth and health. Strain S6 exhibited clear beneficial effects on maize growth, whereas S8 had hardly any effect and is considered as neutral and S7 caused disease symptoms known from *P. ananatis* infections. Therefore, this closely related group of strains represents a promising model to unravel genetic determinants in *P. ananatis* responsible for beneficial and pathogenic effects. Consequently, we functionally characterized the strains by testing for various known plant growth-promoting characteristics as well as for their effect on plant growth, and performed a comparative genome analysis to elucidate genetic features determining the type of plant-microbe interaction.

## Materials and methods

### Maize varieties and seed surface sterilization

Seeds of the maize cultivars (Helmi, Morignon, Pelicon, and Peso) were obtained from local farmers in Seibersdorf, Austria. Maize seeds with no cracks or other visible deformations were surface-sterilized with 70% ethanol for 3 min and 5% sodium hypochlorite for 5 min, and followed by repeated washing with sterile distilled water (3 times for 1 min). The efficacy of surface sterilization was checked by plating 3–5 seeds and aliquots of the final rinse onto 10% tryptic soy agar plates, and incubated for 3 days at 28 ± 1°C. The medium was checked daily for bacterial or fungal growth.

### Isolation of endophytic bacteria from maize seeds

Seed-borne bacteria were isolated following the procedure described by Rijavec et al. (2007) with some modifications. For isolation, 50 surface-sterilized seeds of each cultivar were crushed and blended aseptically in 90 mL of half strength nutrient broth (Difco, Detroit, Michigan) for 5 min. The blend was then incubated at room temperature for 4 h on a rotary shaker (VWR International GmbH, Austria) at 100 r min^−1^. Half strength nutrient broth containing 200 mg/L cycloheximide was inoculated with a series of the incubation mixture (10:1 mL ratio) and further incubated for 4 days on a rotary shaker at room temperature. Aliquots were taken from Erlenmeyer flasks with observed microbial growth and plated onto R2A (Difco, Detroit, Michigan). Plates were incubated at 28°C for 24–48 h. One hundred colonies were picked, and pure cultures were obtained by further streaking on agar plates. Single colonies were picked, inoculated in LB broth and incubated with shaking at 28°C for 24–48 h. Bacterial strains were preserved at −80°C as saturated cultures containing 20% (w/v) glycerol.

### Partial 16S rRNA gene sequencing

For phylogenetic identification of maize seed endophytes we performed partial 16S rDNA (V1 to V3) PCR and sequencing as described by Reiter and Sessitsch (2006). Sequencing was performed by LGC Genomics (Berlin, Germany).

### Preparation of inoculum

Inoculum of the selected strains (S6, S7, S8) were prepared in 100 mL 10% tryptic soy broth in 250 mL Erlenmeyer flasks and incubated at 28 ± 2°C for 48 h in an orbital shaking incubator (VWR International, GmbH) at 180 r min^−1^. The optical density of the broth was adjusted to 0.5 measured at λ 600 nm using spectrophotometer (Gene Quant Pro, Gemini BV, The Netherlands) to obtain an uniform population of bacteria [10^8^–10^9^ colony-forming units (CFU) mL^−1^] in the broth at the time of inoculation.

### Testing the effect of endophytic strains on maize under axenic conditions

Seeds were surface-sterilized by dipping them in 70% ethanol for 3 min and then in a 5% sodium hypochlorite solution for 5 min and subsequently thoroughly washing with sterilized distilled water. The efficacy of surface sterilization was checked by plating seeds, and aliquots of the final rinse onto 10% tryptic soy agar. Samples were considered to be successfully sterilized when no colonies were observed on the tryptic soy agar plates after inoculation for 3 days at 28°C. Surface-disinfected seeds of three maize cultivars (DaSilvie, Kaleo, and Mazurka) were immersed in the bacterial suspensions for 1 h. For the uninoculated control, sterilized tryptic soy broth was used for the seed treatment. Fifteen seeds per treatment were planted in plastic trays with sterilized compost (Blumenerde, COMPO SANA®) and trays were arranged using a randomized design with 3 replications resulting in total number of 45 seeds per treatment. The experiment was conducted for 24 days and data of shoot and root length as well as biomass were recorded.

### Functional characterization of seed endophytic bacteria

#### Phenotypic, physiological, and biochemical characterization

Color and shape of bacterial colonies, growth behavior in different pHs, salt concentrations and C sources as well as aggregate and biofilm formation and motility were tested following the procedures described by Naveed et al. (2014). Biochemical testing of oxidase, catalase, gelatin hydrolysis, and casein hydrolysis activity of the selected strains was performed according to Naveed et al. (2014).

#### Plant growth promoting activities

Strains were tested for activities known to be involved in plant growth regulation and/or rhizosphere competence such as ACC-deaminase activity, auxin production, phosphate solubilization (organic/inorganic P) and siderophore production as well as ammonia, hydrogen cyanide and PHB production as described by Naveed et al. (2014).

#### Cell wall-degrading activities

Bacterial cell wall hydrolyzing activities such as amylase, cellulase, chitinase, lipase, pectinase, phosphatase, protease, and xylanase were screened on diagnostic plates as described by Naveed et al. (2014).

#### Antibiotic resistance of the isolates

Antibiotic resistance was tested individually on tryptic soy agar plates containing the antibiotics ampicillin, cycloheximide, gentamycin, kanamycin, chloramphenicol, rifampicin, spectinomycin, streptomycin or tetracycline respectively at the following concentrations: 25, 50, 75, 100 μg ml^−1^. The plates were incubated at 28 ± 2°C for 5 days and resistance was observed in terms of bacterial growth.

#### Antagonistic activities against plant pathogens

The antagonistic activities of bacterial isolates were screened against plant pathogenic fungi (Fusarium caulimons, Fusarium graminarium, Fusarium oxysporum, Fusarium solani, Rhizoctonia solani, Thielaviopsis basicola) and oomycetes Phytophthora infestans, Phytophthora citricola, Phytophthora cominarum). Antagonistic activity of the bacterial isolates against fungi and oomycetes was tested by the dual culture technique on potato dextrose agar (PDA) and yeast malt agar (YMA) media as described by Naveed et al. (2014).

### Statistical analyses

The data of plant growth parameters and colonization were subjected to analyses of variance (ANOVA). The means were compared with Least Significant Difference (LSD) testing (*p* < 0.05) to detect statistical significance among treatments (Steel et al., 1997). Statistical analyses were conducted using SPSS software version 19 (IBM SPSS Statistics 19, USA).

### Genomic DNA isolation for sequencing

For DNA isolation, the bacterial strains were grown by loop-inoculating one single colony in 5 mL LB broth. The bacterial cultures were incubated at 28 ± 2°C overnight at 180 rpm in a shaking incubator. The overnight cultures were used to inoculate 50 mL fresh LB broth and again incubated at 28 ± 2°C overnight at 180 rpm in a shaking incubator. Bacterial cells were harvested by centrifugation at 4700 rpm for 10 min at 4°C. DNA was extracted from bacterial cell pellets according to the following protocol: The cell pellet was washed with 5 mL lysis buffer (0.1 M NaCl; 0.05 M EDTA, pH 8.0), resuspended in 4 mL lysis buffer containing lysozyme (20 mg mL-1; Roche Diagnostics, Mannheim, Germany) and incubated at 37°C for 20 min. Then 300 μl of 10% sarkosyl was added and placed on ice for 5 min. DNA was extracted with phenol-chloroform-isoamylalcohol (25:24:1, Fluka, Sigma-Aldrich Co.) and re-extracted with chloroform (1:1, Merck, Darmstadt, Germany) followed by precipitation with 0.1 volume of 3 M sodium acetate (pH 5.2) and 2.5 volume of ice-cold absolute ethanol (Merck, Darmstadt, Germany) at −20°C overnight. DNA pellets were washed with 1 ml of 70% ethanol and dissolved in 100 μL TE buffer (10 mM Tris-HCl, pH 7.5; 1 mM EDTA, pH 8·0). DNA was treated with RNase A (final concentration 0.2 gl −1; Invitrogen, Carlsbad, CA) for 90 min at 37°C. DNA quality was analyzed by electrophoresis (80 V) on 0.8% (w/v) agarose gels stained with ethidium bromide. DNA concentration was measured using a NanoDrop 1000 spectrophotometer (Thermo Scientific, Wilmington, DE, USA).

### Genome sequencing, assembly and genome alignment of *P. ananatis* S6, S7, and S8 strains

Genome sequencing of the three strains of *P. ananatis* (S6, S7, and S8) was done by GATC Biotech AG (Konstanz, Germany) using a Roche/454 GS-FLX system. After sequencing, pairwise analysis of average nucleotide identity (ANI) was performed between the *P. ananatis* strains with closed genome sequences and the strains S6, S7, and S8 draft genomes individually as described previously (Goris et al., 2007).

The raw reads from sequencing projects have been deposited at the European Nucleotide Archive (ENA, http://www.ebi.ac.uk/ena/data/view/) under the following project accession numbers: *P. ananatis* S6, PRJEB7511; *P. ananatis* S7, PRJEB7512, and *P. ananatis* S8, PRJEB7513. Genome assemblies are available in ENA under accession numbers CVNF01000001 to CVNF01000077 for *P. ananatis* S6, CVNG01000001 to CVNG01000071 for *P. ananatis* S7 and CVNH01000001 to CVNH01000061 for *P. ananatis* S8. The contigs were assembled using AMOScmp comparative assembler (Pop et al., 2004) and the Roche GS de novo assembler package (Newbler v2.6) in the 454 GS-FLXTM system (http://www.454.com/), indipendently. For AMOScmp assembly, four complete genomes of *P. ananatis* strains (*P. ananatis* AJ13355, *P. ananatis* LMG20103, *P. ananatis* LMG5342 and *P. ananatis* PA13) were used as potential reference genomes. As the assemblies based on *P. ananatis* AJ13355 resulted into highest coverage and mapping quality, this genome was used as reference genome for AMOScmp. The result of quality and coverage control of the assembly of each *P. ananatis* genome sequence was calculated using Qualimap v.1.0 (Garcia-Alcalde et al., 2012). In repetitive regions, such as rRNA operons, the assembly was further evaluated based on the read coverage distribution. Whole genome comparisons between *P. ananatis* S6, S7, and S8 strains were performed using Mauve v.2.3.1 (Darling et al., 2004). In Mauve, the Progressive Mauve algorithm was used to order the contigs against *P. ananatis* AJ13355 as reference genome. Genome assemblies are accessible via http://fileshare.csb.univie.ac.at/pantoea/.

### Overview of *P. ananatis* genomes used in the current study

Five complete genomes of *P. ananatis* strains with different life styles and environmental origin were used in the comparative genomics and phylogenetic analysis. *P. ananatis* PA13 (accession numbers CP003085 and CP003086) is known as a pathogen of rice causing grain and sheath rot (Choi et al., 2012). *P. ananatis* AJ13355 (accession numbers AP012032 and AP012033) shows saprophytic life style and was isolated from soil (Hara et al., 2012). *P. ananatis* LMG20103 (accession number CP001875) is a pathogenic strain causing the severe blight and dieback of *Eucalyptus* (De Maayer et al., 2010). *P. ananatis* LMG5342 (accession numbers HE617160 and HE617161) is an opportunistic human pathogen reported from clinical isolations (De Maayer et al., 2012b). *P. vagans* C9-1 (accession numbers CP002206, CP001893, CP001894, and CP001895) is known as a common plant epiphyte (Smits et al., 2010).

### Phylogenetic analysis

We constructed a phylogenetic tree for *P. ananatis* S6, S7, S8 and the *Pantoea* genomes mentioned above. *P. vagans* C9-1 was included as outgroup. Mauve v2.3.1 (Darling et al., 2004) was used to identify specific and shared SNPs between all compared genomes. The alignments of the genomes were checked manually to eliminate possible false positive SNPs in less conserved regions, particularly if they occur in direct neighborhood of insertions and deletions. The obtained SNPs were filtered based on the position of phylogenetic markers of *P. ananatis* AJ13355 as reference [identified by AMPHORA2; Wu and Scott (2012)] to get the core SNPs of the genome sequences of *P. ananatis* strains. Afterwards, the phylogenetic tree was computed with Geneious 8.0 (Kearse et al., 2012) using 1000 runs for bootstrapping.

### Genome annotation

Gene prediction and annotation were obtained from the in-house ConsPred workflow. ConsPred consists of two phases: *ab initio* as well as homology-based predictions. *Ab initio* predictions are followed by Genemark.hmm (Lukashin and Borodovsky, 1998), Glimmer (Delcher et al., 2007), Prodigal (Hyatt et al., 2010), Critica (Badger and Olsen, 1999) and additional homology based information derived from a BLAST search against the NCBI non-redundant sequence database (NR) (Sayers et al., 2012). Protein domains were predicted by InterProScan (Zdobnov and Apweiler, 2001). For protein sequences without significant hits in NR, functional annotation of protein-coding genes was obtained by a similarity search against the UniProt/SwissProt database (Uniprot consortium, 2009). Non protein-coding elements such as tRNA and rRNA were predicted using tRNAScan and RNAmmer tools, respectively (Lowe and Eddy, 1997; Lagesen et al., 2007). Non-coding RNA genes (ncRNAs) were identified and annotated by a search against RFAM database (Griffiths-Jones et al., 2005).

To check for the completeness of housekeeping genes in the genomes of strains S6, S7, and S8 we used AMPHORA2 (Wu and Scott, 2012) with 31 bacterial phylogenetic marker genes for inferring phylogenetic information.

### Plasmid sequence alignment analysis

To identify the plasmid sequences within the assembled contigs we compared the plasmid sequence of the closest reference genome (*P. ananatis* AJ13355) to the assembly of *P. ananatis* S6, S7 and S8 strains using Mauve v2.3.1 (Darling et al., 2004).

To visualize the coverage of the plasmids in the draft genome sequences, the plasmid sequence of *P. ananatis* AJ13355 were used as reference for comparative circular alignments of the three *P. ananatis* S6, S7, and S8 strains using the BLAST Ring Image Generator (Stothard and Wishart, 2005; Alikhan et al., 2011).

### Comparative genome analyses

#### Identification of orthologous groups

Paralogous and orthologous clusters were identified using OrthoMCL (Li et al., 2003) using the predicted proteomes of seven *P. ananatis* strains (*P. ananatis* AJ13355, *P. ananatis* LMG20103, *P. ananatis* LMG5342, *P. ananatis* PA13 and *P. ananatis* S6, S7, and S8 strains) which initially required an all-vs.-all blastp (E-value cut-off of 1 × 10^−5^). Then the mcl clustering algorithm was used to deduce the relationship between genes.

#### Identification of eukaryotic-like protein domains

To identify eukaryotic-like protein domains (ELDs) in protein sequences in the genomes of strains S6, S7, and S8, those genomes were included in the individual ELD calculation procedure of the Effective web-portal (Jehl et al., 2011). The approach detects protein domains that are present in eukaryotic organisms and significantly enriched in pathogenic and symbiotic compared to non-pathogenic, non-host-associated bacteria. Using default settings, all eukaryotic-like protein domains with an enrichment score greater or equal to 4 were considered for comparison regarding functional differences in *P. ananatis* strains of diverse phenotype.

## Results

### Selection of strains and effects of maize seed endophytes on maize seedling growth

In a previous study, we isolated 90 bacterial strains from seeds of healthy maize plants grown at organic farming fields in Austria. Thirty-seven of these strains shared highest 16S rDNA sequence homology with *P. ananatis* strains (data not shown). Ten strains were randomly selected and tested for effects on seedling growth of maize grown in sterile hydroponic cultures (for a description see Naveed et al., 2014). Along with strains that did not influence maize seedling growth we found one strain with clear detrimental effect and other strains that promoted maize seedling growth (data not shown). One representative of each group was selected and further tested on maize grown in compost. Strain S6 significantly increased seedling growth in all three maize cultivars tested compared to the control (Figure S1; Table 1). Depending on the plant variety root- and shoot-dry biomass was increased up to 47 and 41%, respectively. Root and shoot length was increased up to 57 and 41%, respectively. Strain S8 showed positive effects on plant growth in cultivar DaSilvie only but did not significantly affect growth of the cultivars Kolea and Mazurka (Table 1). In contrast, strain S7 had a negative effect on seedling growth in all the maize cultivars with the effect being significant in DaSilvie and Kolea and less pronounced in Mazurka (Table 1; Figure S1). Apart from reduced biomass S7 treated plants showed white streaks on leaves (Figure S2).

**Table 1:** **Effect of inoculation with seed-associated endophytic bacteria on root/shoot length and biomass of maize seedlings**.

**Strains**	**DaSilvie**	**Kaleo**	**Mazurka**	**DaSilvie**	**Kaleo**	**Mazurka**
	**Root length (cm)**			**Shoot length (cm)**		
Control	16.67 fgh^a^	15.67 gh	19.00 ef	27.67 bcd	24.67 ef	25.33 def
*P. ananatis* S6	25.67 ab	24.50 bc	27.33 a	34.66 a	34.97 a	34.83 a
*P. ananatis* S7	16.33 fgh	15.00 h	17.67 fgh	26.67 cde	26.33 cde	23.33 f
*P. ananatis* S8	20.67 de	18.00 fg	21.38 de	30.00 b	28.33 bc	27.00 bcd
	**Root dry biomass (mg)**			**Shoot dry biomass (mg)**		
Control	20.98 cde	20.49 de	22.78 bcd	229.31 e	224.38 e	248.75 bcd
*P. ananatis* S6	29.57 a	30.09 a	30.51 a	323.11 a	324.00 a	330.42 a
*P. ananatis* S7	18.78 ef	17.23 f	21.24 cde	199.29 f	191.18 f	232.45 de
*P. ananatis* S8	24.02 b	22.03 bcd	23.39 bc	256.45 bc	241.25 cde	262.89 b

a *Means sharing same letter(s) do not differ significantly at P = 0.05*.

### Functional characterization of maize seed isolates based on *in vitro* assays

A range of activities known to contribute to plant growth promotion, stress tolerance or biocontrol was tested. The results of functional characterization are summarized in Table 2. All strains exhibited ACC-deaminase activity and showed auxin, NH_3_ and siderophore production (qualitative). All three strains showed P-solubilization and were able to produce AHL and PHB. S6, S7, and S8 behaved similar in tests for motility and chemotaxis as well as the biochemical characters mentioned in Table 2. No strain produced EPS in our assays. Lipase, pectinase, phosphatase and xylanase activity was detected in all strains, whereas none of the strains showed amylase, cellulose, chitinase or protease activity. Strain S6 showed *in vitro* antagonistic activity against all bacterial pathogens tested but *F. solani*. Strain S7 inhibited growth of *F. oxysporum, T. basicola*, and *P. citricola* in our assays, whereas strain S8 negatively affected growth of *F. graminarium, F. oxysporum, R. solani*, and *P. citricola*.

**Table 2:** **Physico-chemical and growth-promoting characteristics of maize seed-borne endophytic bacteria**.

**Characteristics**	***P. ananatis* S6**	***P. ananatis* S7**	***P*. *anantis* S8**
**PHENOTYPIC CHARACTERIZATION**			
Colony color	Yellow	Yellow	Yellow
Colony morphology	Round	Round	Round
**BACTERIAL GROWTH CONDITIONS**			
**Temperature**			
4°C	+	+	+
42°C	−	−	−
**NaCl**			
2%	+	+	+
6%	+	+	+
**pH:**			
5	+	+	+
12	+	+	+
**MOTILITY/CHEMOTAXIS^a^**			
Swimming	+	+	+
Swarming	++	+	+
Twitching	+	+	+
**Biofilm formation**			
OD (600 nm)	0.95 ± 0.08	0.89 ± 0.07	0.92 ± 0.06
Biofilm (595 nm)	0.08 ± 0.01	0.07 ± 0.01	0.06 ± 0.01
Aggregate stability (%)	32.61 ± 2.13	28.61 ± 1.93	30.61 ± 2.01
**BIOCHEMICAL CHARACTERIZATION^b^**			
Catalase	+	+	+
Oxidase	−	−	−
Casein	−	−	−
Gelatin	3.5 ± 0.15	2.9 ± 0.10	3.2 ± 0.12
Methanol	−	−	−
Ethanol	−	−	−
**GROWTH PROMOTING CHARACTERIZATION^a^**			
ACC-deaminase activity	+	+	+
**Auxin production (IAA equivalent μg mL^−1^)**			
Without L-TRP	0.87 ± 0.55	0.68 ± 0.52	0.78 ± 0.54
With L-TRP	32.67 ± 3.17	27.45 ± 2.89	30.89 ± 3.17
**P-solubilization (Inorganic/organic P)**			
Ca_3_(PO_4_)_2_	1.6 ± 0.10	1.2 ± 0.14	1.4 ± 0.14
CaHPO_4_	1.5 ± 0.08	1.0 ± 0.06	1.2 ± 0.08
Ca-Phytate	2.5 ± 0.11	2.0 ± 0.10	2.3 ± 0.11
Na-Phytate	1.4 ± 0.06	0.9 ± 0.02	1.0 ± 0.06
Exopolysaccharide	−	−	−
HCN production	−	−	−
NH_3_ production	+	+	+
Siderophore	−	−	−
AHL	+	+	+
PHB	+	+	+
**ENZYME HYDROLYZING ACTIVITY^a^ (COLON DIAMETER CM)**			
Amylase	−	−	−
Cellulase	−	−	−
Chitinase	−	−	−
Lipase	2.2 ± 0.09	1.8 ± 0.08	2.0 ± 0.09
Pectinase	1.5 ± 0.11	1.2 ± 0.04	1.0 ± 0.05
Phosphatase	1.6 ± 0.08	1.3 ± 0.07	1.0 ± 0.08
Protease	−	−	−
Xylanase	1.3 ± 0.09	0.8 ± 0.02	1.0 ± 0.06
**ANTIBIOTIC RESISTANCE (μg mL^−1^)**			
Ampicillin	−	−	−
Gentamycin	−	−	−
Kanamycin	−	−	−
Chloramphenicol	−	−	−
Rifampicin	−	−	−
Spectinomycin	−	−	−
Streptomycin	−	−	−
Tetracycline	−	−	−
**ANTI-FUNGAL ACTIVITY (COLON DIAMETER cm)**			
*F. caulimons*	2.0 ± 0.05	−	−
*F. graminarium*	1.2 ± 0.04	−	1.0 ± 0.04
*F. oxysporum*	1.0 ± 0.03	1.0 ± 0.03	1.0 ± 0.03
*F. solani*	−	−	−
*R. solani*	1.8 ± 0.07	−	1.5 ± 0.06
*T. basicola*	1.2 ± 0.05	1.2 ± 0.05	−
**ANTI-OOMYCETE ACTIVITY**			
*P. infestans*	3.4 ± 0.11	−	−
*P. citricola*	3.5 ± 0.09	3.0 ± 0.09	3.0 ± 0.12
*P. cominarum*	2.8 ± 0.08	−	−

a *Results in characterization table are of 4–6 replicates*.

b *−, absent; +, present*.

### Genome sequences of *P. ananatis* strains S6, S7, and S8

Genomic DNA of strains S6, S7, and S8 was sequenced and the generated raw reads represented 230, 76, and 79 million bases respectively (Table 3). The number of sequenced reads varied from 570,490 in strain S6 with an average length of 406 bp to 174,500 and 179,051 in S7 and S8 respectively, with an average length of 441 bp in both strains.

**Table 3:** **Genome characteristics of sequencing and assembly of three strains of *P. ananatis* S6, S7, and S8**.

**Species**	**Strain**	**Total nucleotides (bp)**	**Total reads**	**Average length**
**SEQUENCING STATISTICS**				
*P. ananatis*	S6	231,806,398	570,490	406
*P. ananatis*	S7	76,917,000	174,500	441
*P. ananatis*	S8	79,039,900	179,051	441

**Table:** 

**Species**	**Strain**	**# Contigs**	**N50**	**Total size**	**Assembly Score**	**Average Coverage**
**COMPARATIVE ASSEMBLY STATISTICS (AMOScmp)**						
*P. ananatis*	S6	93	127341	4361793	5972420241	43.08
*P. ananatis*	S7	92	134747	4553649	6669462411	13.62
*P. ananatis*	S8	63	178470	4618012	13082168280	14.38

The pairwise comparision of average nucleotide identity (ANI) of the draft genomes of strains S6, S7, and S8 with the *P. ananatis* AJ13355 genome showed that the similarity of the analyzed strains and strain AJ13355 exceeds 99% (Supplementary Table 1).

Comparative sequence assembly was performed by AMOScmp program (Pop et al., 2004) as a conservative method that uses the most similar available complete genome sequence as a reference to assemble the 454 reads (Table 3). The *P. ananatis* S6, S7, and S8 draft genomes consist of 93, 92, and 63 contigs, respectively, and range from 4.3 to 4.6 Mb in length.

De novo assembly resulted in almost the same coverage assembly but less assembly score and N50 value in comparison to AMOScmp assembler.

**Figure 1:**
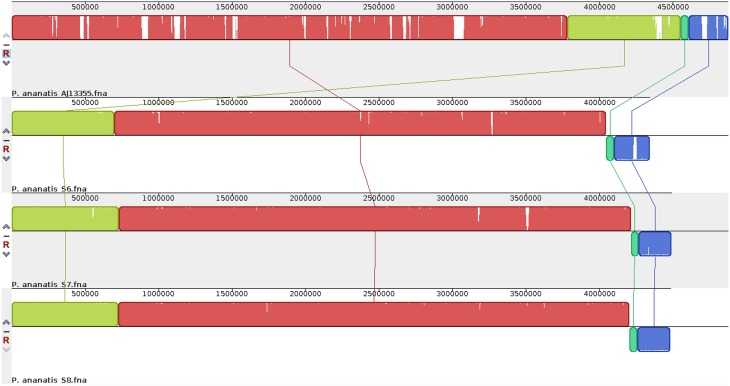
**Genome-scale comparison for draft genome sequences of the three *P. ananatis* strains (S6, S7, and S8) and complete genome sequence of *P. ananatis* AJ13355**. Homologous DNA regions among the strains are marked by the same colored blocks, while gaps correspond to non-homologous regions. The figure was generated using nucleotide sequences of the genomes using Mauve v2.3.1.

**Figure 2:**
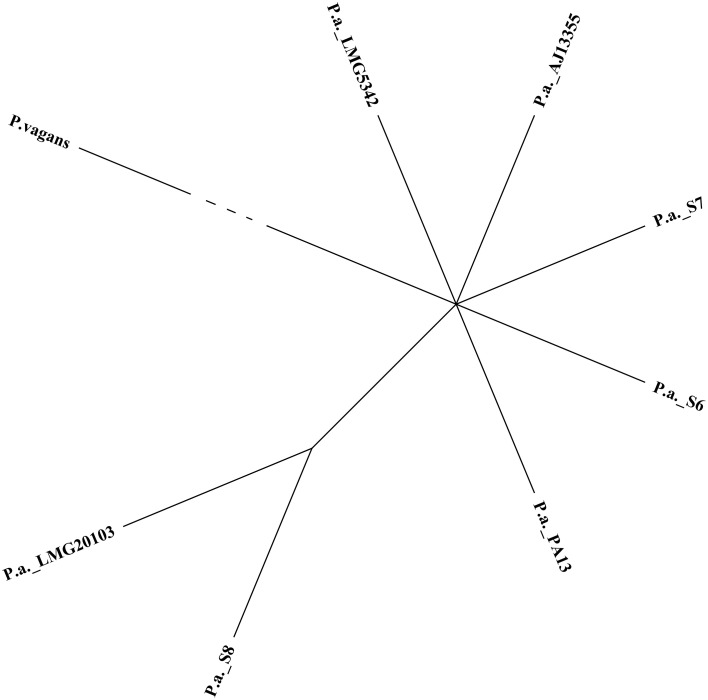
**Phylogenetic trees of three strains of *P. ananatis* S6, S7 and S8 with four closed genomes from *Pantoea* genus**. *Pantoea vagans* was included as an out group (edge has been shortened).

The comparison of draft genome assembly for *P. ananatis* S6, S7, and S8 against *P. ananatis* AJ13355 as reference genome are shown in Figure 1, illustrating a higher degree of genome conservation among the strains S6, S7, S8, as compared to *P. ananatis* AJ13355 (Figure 1). Phylogenetic analysis revealed a close relationship between strains S6, S7, S8, and the other four genomes of *P. ananatis* in comparison to *P. vagans* (Figure 2).

### Genome annotation of *P. ananatis* S6, S7, and S8 strains

The genome annotation of *P. annanatis* S6, S7, and S8 resulted in different numbers of protein-coding genes. The genome of strain S6 consists of 4.375 predicted coding sequences (CDSs), while S7 and S8 contain 4.516 and 4.528 predicted CDSs, respectively (Table 4). Seven 16S rRNA, seven 23S rRNA and eight 5S rRNA genes are encoded in each of the *P. annanatis* strains. In total all tRNA genes for 33 different anticodons were found in all *P. annanatis* strains. The results of annotation analysis of three *P. ananatis* S6, S7, and S8 strains and reannotation of *P. ananais* strains (*P. ananatis* AJ13355, *P. ananatis* LMG20103, *P. ananatis* LMG5342, and *P. ananatis* PA13) are summarized in Table 4.

**Table 4:** **Comparison of (A) draft genome annotation of three *P. ananatis* S6, S7, and S8 strains and (B) re-annotation of four complete genome of *P. ananatis* strains**.

	**Species**	**Strain**	**GC content (%)**	**#CDS**	**tRNA**	**rRNA**			**ncRNA**	**Pseudogenes**
						**5S**	**16S**	**23S**		
(A)	*P. ananatis*	S6	54	4375	69	8	7	7	144	11
	*P. ananatis*	S7	54	4516	68	8	7	7	143	13
	*P. ananatis*	S8	54	4528	68	8	7	7	142	11
(B)	*P. ananatis*	AJ13355	54	4977	78	8	7	7	167	21
	*P. ananatis*	LMG5342	53	5010	77	8	7	7	154	12
	*P. ananatis*	LMG20103	54	4715	70	8	7	7	154	24
	*P. ananatis*	PA13	54	5038	83	8	7	7	167	13

The fact that tRNA genes for all essential amino acids, the 16S rRNA gene and 31 housekeeping genes were found in the draft genomes of strains S6, S7, and S8 indicates that the genomes are close to complete. Moreover, the overall pattern of distribution of housekeeping genes and the gene copy number are identical to other members of the *Enterobacteriaceae* family.

To verify the sequence quality generated by 454 sequencing technology we identified putative pseudogenes represented by frameshifts in the draft genomes of *P. ananatis*. The low number of pseudogenes in the genomes of strains S6, S7, and S8 (11, 13, and 11 respectively) indicated that the genome draft has sufficient quality for further comparative genomics analysis (Table 4).

### Plasmid sequence alignment analysis

Five, six, and seven contigs in *P. ananatis* S6, S7, and S8 genome sequences, respectively, were homologous with the plasmid sequence of *P. ananatis* AJ13355 (Supplementary Table 2). In total, 287, 271, and 276 genes were identified in the plasmid contigs of strains S6, S7, and S8. The core factors identified on the large universal *Pantoea* plasmid LPP-1 (De Maayer et al., 2012a) such as genes coding for thiamine biosynthesis proteins (*thiOSF*), pigment biosynthetic protiens (*crtEXYIBZ*), arbutin/cellobiose/salicin transport and catabolism components (*ascBFG*), malate:quinone oxidoreductase (*mqo*), 1,3-diaminopropane production (dat, ddc) and branched-chain amino acid transport protein (*azlDC*) are present on the plasmid sequences of *P. ananatis* S6, S7, and S8 (Supplementary Table 1).

**Figure 3:**
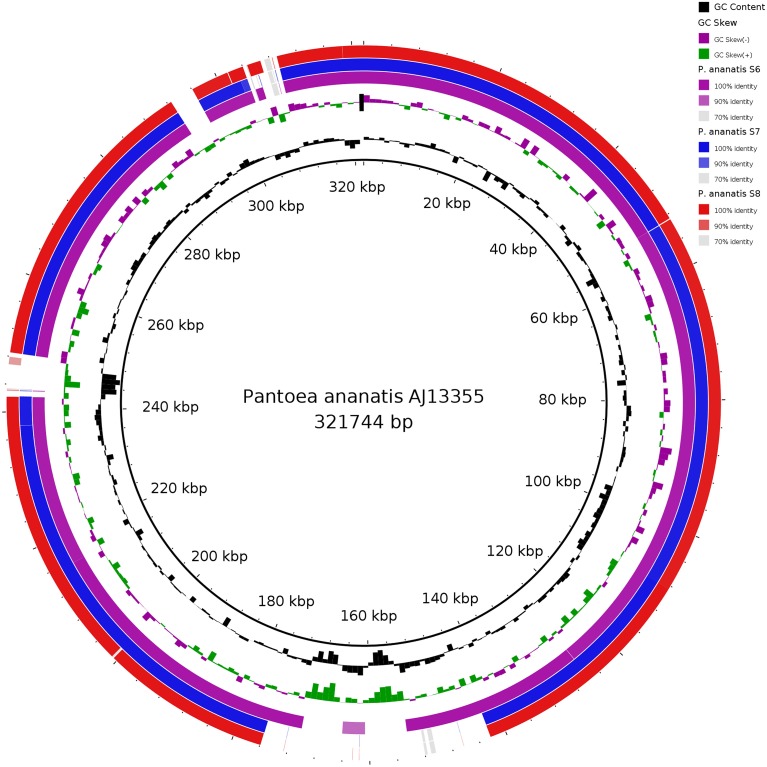
**Comparison of the circular genome map of plasmid sequences of three *P. ananatis* S6, S7, and S8 genome structures with the known *P. ananatis* AJ13355 plasmid sequence as reference genome using blast ring image generator (BRIG)**. The inner circle shows the scale (bp). The first and the second rings show the GC content (black) and GC skew (purple/green), respectively, with respect to the reference genome. The 3rd, 4th, and 5th rings show BLAST comparisons of *P. ananatis* strains S6, S7, and S8 plasmid sequences, respectively.

Comparative circular blast alignments of the plasmid sequences in Figure 3 shows high homology between plasmid sequences of *P. ananatis* S6, S7, and S8 as compared to the *P. ananatis* AJ13355 plasmid sequence (Stothard and Wishart, 2005; Alikhan et al., 2011) (Figure 3).

### Comparative genomics analysis

**Figure 4:**
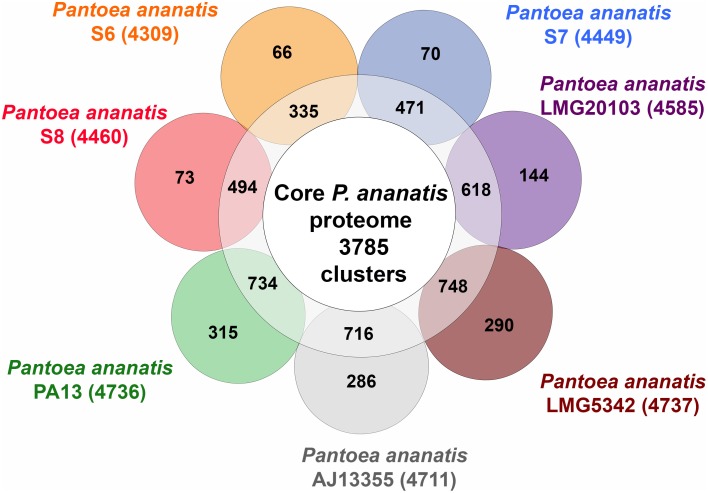
**Clusters of orthologous gene families in seven *P. ananatis* strains identified by OrthoMCL**. The inner circle shows the core proteome shared between all strains. The numbers of gene clusters shared between specific strains are shown in the ring. The specific proteins for each strain are indicated in each of the outer circles. The numbers outside the Venn diagram show the total number of genes (in parentheses) for each strain.

**Figure 5:**
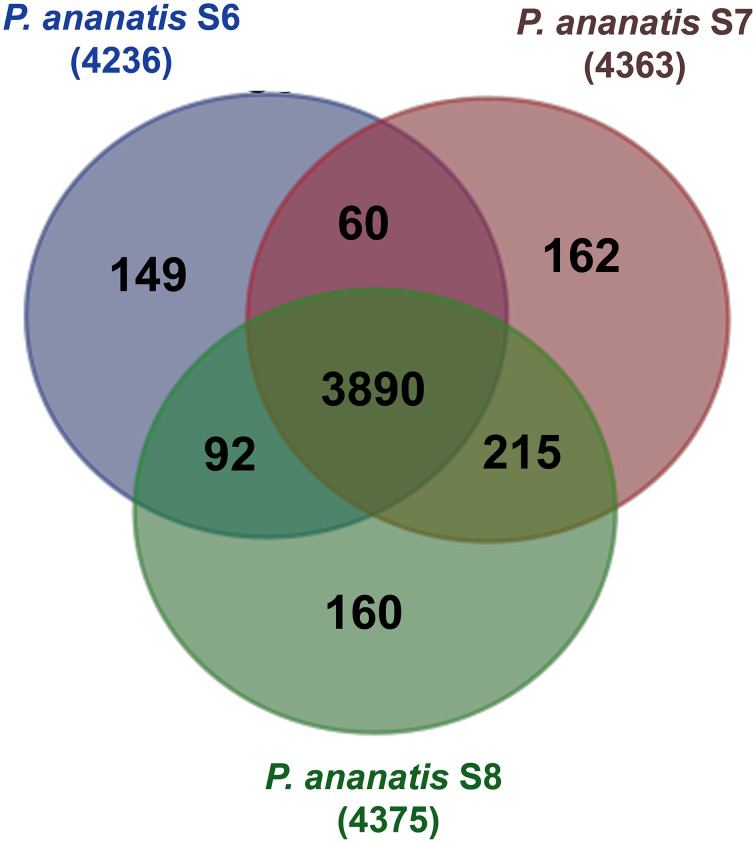
**Venn diagram of OrthoMCL cluster distribution across three *P. ananatis* S6, S7, and S8 strains identified by OrthoMCL**. The number of core proteome clusters, gene families shared between the species and the specific proteins for each strain is indicated in each of the components. The numbers outside the Venn diagram show the total number of genes (in parentheses) for each strain.

To identify the core *P. ananatis* genome, we clustered orthologous groups from genes predicted in the seven *P. ananatis* genomes of this study (*P. ananatis* AJ13355, *P. ananatis* LMG20103, *P. ananatis* LMG5342, *P. ananatis* PA13 and strains S6, S7, S8) using OrthoMCL (Li et al., 2003). Of the total 33,159 protein-coding genes in all *P. ananatis* strains, 31,987 genes clustered into 4959 gene families. Out of these, 27,578 genes representing 3785 gene families, were common to all *P. ananatis* strains, hereafter referred to as the core *P. ananatis* proteome (Figure 4). Fifty-three clusters were shared between *P. ananatis* S6 and S7. *P. ananatis* S7 and S8 have 207 clusters in common while *P. ananatis* S6 and S8 shared 79 clusters (Figure 5).

### Gene functional classification of *P. ananatis* strains

To understand the functions of shared and specific genes between the *P. ananatis* strains, we analyzed the functional categories of the respective *P. ananatis* gene clusters based on the NOG annotations (Jensen et al., 2008).

As expected, the core *P. ananatis* genes were categorized in functions involved in metabolism, cellular processes and signaling activity, information storage and processing (Supplementary Tables 3, 5). The beneficial *P. ananatis* S6 specific genes encode proteins with putative functions in metabolism, signal transduction and information storage and processing. Whereas, pathogenic *P. ananatis* S7 specific genes were specifically involved in cell cycle control, cell division, chromosome partitioning and amino acid transport. The commensal *P. ananatis* S8 specific genes were responsible for transcription and amino acid transport (Supplementary Tables 4, 6).

### Functional annotation of *P. ananatis* strains

**Figure 6:**
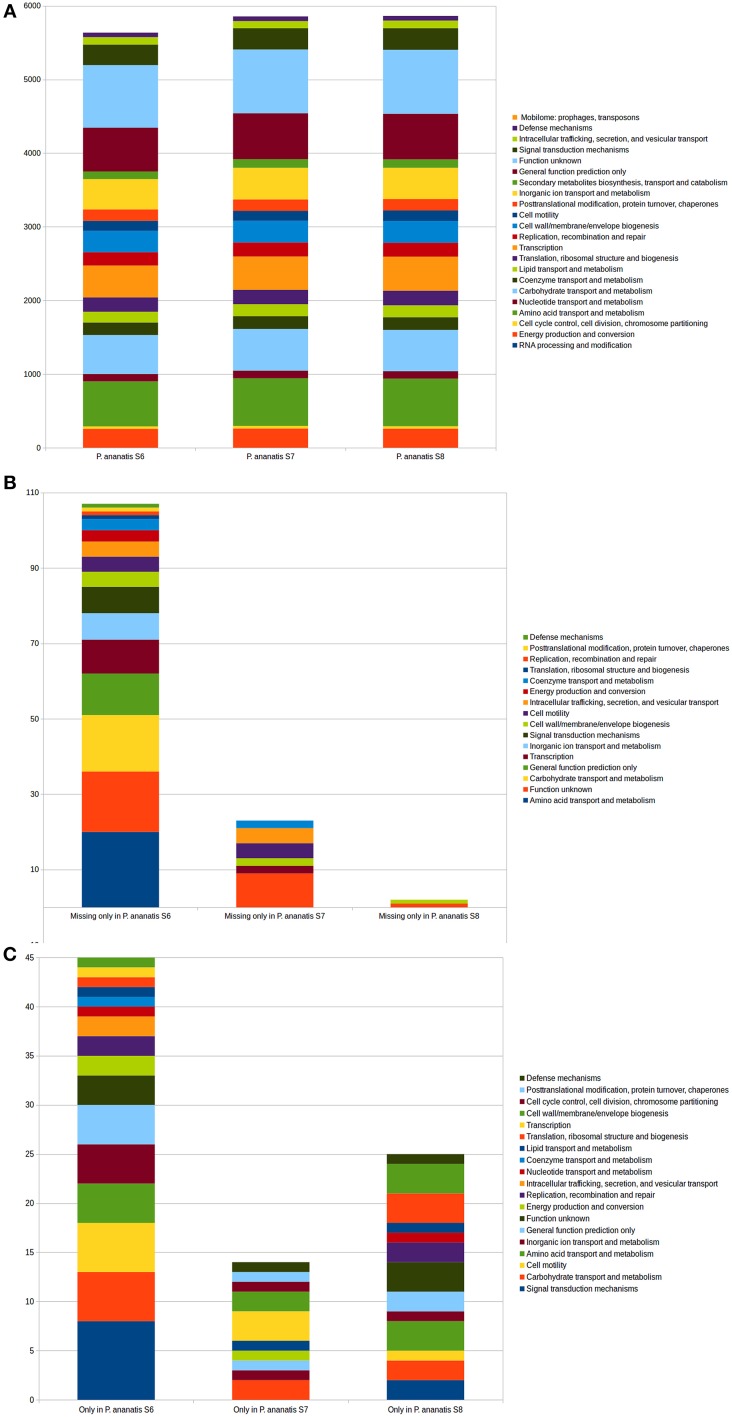
**Functional COG categories in the genomes of the three *P. ananatis* strains S6, S7, and S8**. **(A)** Comparison of the COG categories in the genomes of the three *P. ananatis* strains S6, S7, and S8. **(B)** The COG categories that present in two of the *P. ananatis* strains but are absent in the third strain (S6, S7, or S8). **(C)** The COG categories existing in only one of the *P. ananatis* strains (S6, S7, or S8).

Functional annotations of orthologous groups on the predicted proteomes of S6, S7, and S8 and the published *P. ananatis* genomes revealed functions that were common within all the genomes. This analysis also indicated gene families that cause differences among the strains on the functional level. The distribution of genes in COG functional categories is shown in Figure 6.

**Type IV pilus biogenesis proteins** such as *PilNQCWTZ*, type IV pilus secretin *PilQ*, pili assembly chaperone and prepilin type IV endopeptidase were found in the core proteome of *P. ananatis* strains (Supplementary Table 5). Interestingly, two genes related to pili assembly chaperon and fimbrial-type adhesion (the uncharacterized fimbrial chaperone *YhcA* and *F17a-A* fimbrial protein) were found in all *P. ananatis* strains but missing from beneficial *P. ananatis* S6 and two other genes related to the pili (fimbrial chaperone *YfcS* and chaperone protein *PapD*) were absent in pathogenic *P. ananatis* S7 but found in all other *P. ananatis* strains (Supplementary Table 7; Figure 6B).

**Transposases related proteins** such as tyrosine recombinase *XerD*, tyrosine recombinase *XerC* and site-specific recombinase *XerD* were identified in the core proteome of all *P. ananatis* strains. The only difference seen was the*YhgA*-like transposase that was found only in the commensal *P. ananatis* S8 (Supplementary Tables 5, 6; Figures 6A,C).

**Virulence associated genes on mobile genetic elements** showed that phage/bacteriophage related proteins such as bacteriophage P2 (*GpU)*, bacteriophage tail protein *Gp41* and phage tail tape measure protein are present in *P. ananatis* S7 and S8 and all other *P. ananatis* strains but do not have orthologs in the beneficial *P. ananatis* strain S6. The bacteriophage T7, Gp4, DNA primase/helicase is presented only in commensal *P. ananatis* S8 strain. Orthologous for integrases were not found in the beneficial strain S6 but were presented in the other strains (Supplementary Tables 6, 7; Figures 6B,C).

**The chemotaxis related proteins** such as chemotaxis methyl-accepting receptor (*CheR*) and chemotaxis proteins (*CheVWY*) were identified in the core proteome of the *P. ananatis* strains. The methyl-accepting chemotaxis signaling proteinI *TSR* is missing in the beneficial *P. ananatis* S6 strain but this strain contains the methyl-accepting chemotaxis signaling protein (*MCP*) which has the same activity in transducing the signal to downstream signaling proteins in the cytoplasm (Supplementary Tables 5, 6; Figures 6A,C).

**The orthologous groups that are related to flagellar structures** in the core *P. ananatis* proteome consists of flagella basal body P-ring formation proteins FlgAC; flagellar assembly proteins *FliH*; flagellar basal body rod protein components *FlaE, FlgJ*; flagellar hook-basal body complex proteins *FliELK, FlgCK* and flagellar biosynthesis proteins *FlhAQRO*. Other main flagellar related proteins are *FliJ, FlhE* flagellar motor protein MotA/MotB and *FliNGMSTZ* identified in the core proteome of *P. ananatis* strains (Supplementary Table 5; Figure 6A).

**Gene families for T6SS loci** were found on the core proteome of all seven *P. ananatis* strains investigated in this study. These common genes encoding *DotU* (COG2885), ATPase *ClpV1* (COG0542), FHA domain-containing protein (COG3456), *IcmF* (COG3523), lipoprotein *SciN* (COG3521), lysozyme-related protein (*impF*) (COG3518), OmpA/MotB domain (COG3455), T6SS RhsGE-associated *Vgr* family subset (COG3501), T6SS-associated *BMAA0400* (COG3913), T6SS -associated *ImpA* (COG3515) and *Hcp1* (COG3157) (Supplementary Table 5; Figure 6A). Our analysis showed also that the outer component of the T6SS, which consist of two proteins, *VgrG* (COG3501) and *Hcp* (COG3157) have also been identified as secreted effectors of the T6SS in some of the *P. ananatis* strains (Supplementary Table 7; Figure 6B). The effector protein genes *hcp*1, *hcp*1_2, and *hcp*1_3 loci are presented in six *P. ananatis* strains but absent in the pathogenic strain *P. ananatis* S7 (Table 5). The *HcpC* as major exported protein is missing in commensal *P. ananatis* S8 and pathogenic strains *P. ananatis* S7 and LMG5342, however it was present in the beneficial S6 strains, *P. ananatis* AJ13355 and pathogenic strains of *P. ananatis* PA13 and *P. ananatis* LMG20103 (Supplementary Table 7; Figure 6B).

**Table 5:** **Hemolysin co-regulated effector proteins (Hcp) presented in the Type VI secretion system identified in orthologous clusters of *P. ananatis* strains**.

**P. *ananatis* Strains**	**T6SS hemolysin co-regulated effector proteins (Hcp)**			
	Hcp1 (PAGR_1583)^*^	Hcp1_2 (PAGR_1584)^*^	**Hcp1_3 (IPR008514)**	HcpC (PAGR_3636)^*^
S6	BN1182_BN_00010	BN1182_BN_00910	BN1182_BN_00920	BN1182_CY_00040
S7	–	–	–	–
S8	BN1184_BC_00200	BN1184_BC_01090	BN1184_BC_01100	–
AJ13355	PantAJ13_A_20550	PantAJ13_A_21490	PantAJ13_A_21500	PantAJ13_B_01630
PA13	PantPA13_B_18870	PantPA13_B_18000	PantPA13_B_17990	PantPA13_B_41060
LMG20103	PantLMG20_A_26140	PantLMG20_A_27020	PantLMG20_A_27030	PantLMG20_A_46190
LMG5342	PantLMG53_A_18720	PantLMG53_A_17820	PantLMG53_A_17810	–

* Hcp locus tag PAGR-* are reported in Shyntum et al. (2014).

### Eukaryotic-like protein domains in *P. ananatis* strains

We identified eukaryotic-like protein domains (ELDs) in strains S6, S7, and S8 by applying the prediction framework of the Effective web-portal (Jehl et al., 2011). The prediction assigns a eukaryotic-like domain enrichment score (ELD score) to each protein domain, reflecting the maximal enrichment of that domain in any pathogen or symbiont compared to the background frequency of the protein domain in non-pathogenic, non-host-associated bacteria. A high ELD score equals strong enrichment of the protein domain in pathogenic/symbiotic bacteria and suggests an important functional role of the secreted protein in the interaction with the host cell. All ELDs with a significant ELD score greater or equal to 4 were considered to investigate the genomic variance of *P. ananatis* strains S6, S7, and S8 that cause different phenotypes in the host plant.

In summary, 29 different ELDs were predicted (Table 6). The majority, i.e., 26 ELDs are shared between all three genomes, supporting the assumption of a high average functional similarity of effector proteins. One eukaryotic-like protein domain, the tRNA delta-isopentenylpyrophosphate (IPP) transferase domain (PF01715) was exclusively found in the genome of the beneficial maize seed strain *P. ananatis* S6. IPP transferases are involved in the modification of tRNAs and convert A(37) to isopentenyl A(37). Another one was unique in the pathogenic strain S7 and contains the C terminal part of a GMP synthase (PF00958). This enzyme belongs to the family of ligases and is involved the biosynthesis of the nucleic acid guanine. A eukaryotic-like domain containing the signature of the collagen-binding domain of bacterial collagenases (PF12904) was found in S7, S8 and all other *P. ananatis* genomes but was absent in S6.

**Table 6:** **Differences of eukaryotic-like protein domain (ELD) enrichment in *P. ananatis* strains of diverse phenotype**.

	**Pfam ID**	**Domain description**	ELD Score^*^
Only in pathogenic *P. ananatis* S7	PF00958	GMP synthase C terminal domain	7
Only in beneficial *P. ananatis* S6	PF01715	IPP transferase	5
missing only in beneficial *P. ananatis* S6	PF12904	Putative collagen-binding domain of a collagenase	6
Shared in all *P. ananatis* S6, S7, and S8 strains	PF14328	Domain of unknown function (DUF4385)	–
	PF14145	YrhK-like protein	–
	PF13718	GNAT acetyltransferase 2	–
	PF13347	MFS/sugar transport protein	–
	PF10685	Stress-induced bacterial acidophilic repeat motif	–
	PF09825	Biotin-protein ligase N terminal	–
	PF09330	D-lactate dehydrogenase membrane binding	–
	PF08351	Domain of unknown function (DUF1726)	–
	PF08125	Mannitol dehydrogenase C-terminal domain	–
	PF07798	Protein of unknown function (DUF1640)	–
	PF07350	Protein of unknown function (DUF1479)	–
	PF06500	Alpha/beta hydrolase-unknown function- DUF1100	–
	PF05870	Phenolic acid decarboxylase (PAD)	–
	PF05704	Capsular polysaccharide synthesis protein	–
	PF05433	Glycine zipper 2TM domain	–
	PF05127	Helicase	–
	PF03825	Nucleoside H+ symporter	–
	PF02551	Acyl-CoA thioesterase	–
	PF01306	LacY proton/sugar symporter	–
	PF01232	Mannitol dehydrogenase Rossmann domain	–
	PF01204	Trehalase	–
	PF01116	Fructose-bisphosphate aldolase class-II	–
	PF00625	Guanylate kinase	–
	PF00328	Histidine phosphatase superfamily (branch 2)	–
	PF00294	pfkB family carbohydrate kinase	–
	PF00070	Pyridine nucleotide-disulphide oxidoreductase	–

* *The domains without score have different scores for each P. ananatis strains*.

## Discussion

The genus *Pantoea* comprises bacteria that are frequently associated with eukaryotic hosts such as plants but strains, even those belonging to the same species (such as *P. ananatis*), have different type of interactions with their host ranging from pathogenicity to mutualism (De Maayer et al., 2014). In our study we showed that genetically closely related *P. ananatis* strains with different effects on plant growth colonize maize seeds.

In our study, the maize seed endophyte *P. ananatis* S6 showed clear beneficial effects on maize growth, while strain S7 induced weak pathogenicity symptoms. *P. ananatis* S8 had hardly any effect and can be considered as commensal. The pan genome of eight *P. ananatis* genomes indicated as open pan genome that they can colonize and exploit several different environmental niches by De Maayer et al. (2014). As three *P. ananatis* strains (S6, S7, and S8) are also capable to colonize inside maize seeds and interact with their host, we can expect that the pan genome of these strains can be defined as open pan genome.

Our comparative analysis showed that an average of 85–87% of CDSs predicted for each individual strain of *P. ananatis* S6, S7, and S8 have orthologs encoded by the genomes of the other strains (*P. ananatis* AJ13355, *P. ananatis* LMG20103, *P. ananatis* LMG5342 and *P. ananatis* PA13). These results suggest that the core genomes of strains S6, S7, and S8 strains are highly conserved (Figure 1). Despite the overall high degree of similarity between the core genomes of the three maize seed endophytes, we found differences in transposase/integrases/phage related genes, type VI secretion system, and eukaryotic-like protein domains. Similarly, the analysis of the open pan-genome of eight sequenced genomes of *P. ananatis* indicated that between 89.3 and 95.7% of the proteins are common between all strains and they are important for metabolism and cellular processes (De Maayer et al., 2014).

Genes of the accessory genome of selected *P. ananatis* strains analyzed by De Maayer et al. (2014) encoded mainly poorly characterized proteins including transposases, integrases, and mobile genetic elements. The role of horizontal gene transfer in the diversification of *P. ananatis* strains was suggested (De Maayer et al., 2014). Similarly, phage related genes were reported to have a significant role in transferring pathogenicity factors to their bacterial host and thereby to affect bacterial evolution (Lima-Mendez et al., 2008). Due to the differences found in regard mobile genetic elements such as integrase genes, transposase genes and phage related genes, our study confirms a potential role of these elements in the diversification of related strains colonizing the same ecological niche. An over-representation of transposase genes and mobile elements also indicates the genomes' potential for acquisition of novel functions. The reduced number of mobile elements in *P. ananatis* S6 on the other hand could indicate high stability of its genome, implying good adaption to the habitat.

De Maayer et al. (2012a) proposed the Large Pantoea Plasmid (LLP-1) as genetic determinant of niche adaption and functional diversification of the genus *Pantoea*. All three maize seed endophytes S6, S7, and S8 contain a LLP-1 plasmid and no differences in LPP-1 related genes were found between the genomes of these strains and the core genome of *P. ananatis*. Our analysis revealed further that genes encoding the pigment biosyntetic (*CrtEXYIBZ*) and thiamine biosynthesis (*ThiOSGF*) proteins are present on the plasmid of *P. ananatis* S6, S7, and S8 (Supplementary Table 1). These genes are among those genes identified by De Maayer et al. (2014) to be specific for plant-associated bacteria (PAB) among *P. ananatis*. In addition, the core proteome of the maize seed *P. ananatis* strains contains PAB-specific CDs with prediction functions in metabolism and transport of carbohydrates, iron uptake and metabolism, and carbon, nitrogen and energy sources (De Maayer et al., 2014). In conclusion, our findings support the concept of functional diversification of the species *P. ananatis* proposed by De Maayer et al. (2014).

The T6SS is one of the most studied secretion system in *P. ananatis* (Coutinho and Venter, 2009; De Maayer et al., 2011; Shyntum et al., 2014). Three T6SS loci (T6SS-1, -2, and –3) have been described in *P. ananatis* strains, translocating effectors into the host plant (De Maayer et al., 2011; Shyntum et al., 2014). The T6SS-1 locus is found on the genomes of all *P. ananatis* strains, while T6SS-2 is restricted to pathogenic strains of *P. ananatis*. The presence of T6SS-1 and T6SS-2 in both pathogenic and non-pathogenic *P. ananatis* strains support the idea that the T6SS itself is not necessarily a determinant of pathogenicity and could play a role in competition against other microorganisms, fitness or niche adaptation (Weber et al., 2009; English et al., 2012; Shyntum et al., 2014). T6SS-3 was found to be mainly restricted to *P. ananatis* AJ13355, *P. ananatis* LMG 20103, and *P. ananatis* PA4 (De Maayer et al., 2014).

Beside the T6SS loci related genes VgrG and Hcp genes are present in the maize seed *P. ananatis* strains. The VgrG genes were found in S6, S7, and S8, whereas differences were seen in the presence of hemolysin co-regulated effector proteins (Hcp) between these three strains. A recent study showed that three *hcp* genes exist in *P. ananatis* strains comprising *hcp*-1, *hcp*-2 (having homologs in all sequenced strains of *P. ananatis*) and *hcp*-3 genes (found in *P. ananatis* PA13) (Shyntum et al., 2014). The *hcp*-3 gene is highly divergent from *hcp*-1, *hcp*-2, and the T6SS associated *hcp* genes (Shyntum et al., 2014). The plant-beneficial strain S6 has orthologs with all *hcp* genes identified in the orthologous gene families, while plant-pathogenic strain S7 has no orthologs for *hcp* genes. HcpC is presented in all *P. ananatis* strains but it is missing from S7 and S8 strains. This Hcp protein is located on the plasmid sequence of this strain. Paralogs of *hcp* influence bacterial motility, protease production and biofilm formation (Sha et al., 2013). A potential role of *Hcp* and V*gr*G proteins in host interaction is not described. As all *hcp* genes are present in other *P. ananatis* strains (ranging from pathogenic to saprophytic life style), the *hcp* genes in in the beneficial *P. ananatis* strain S6 might not be responsible for the differences in the phenotype of plant-microbe interaction of the three maize seed strains S6, S7, and S8.

The analysis of effector candidates containing eukaryotic-like protein domains (ELDs) revealed varying molecular repertoire in the genomes of the three maize seed *P. ananatis* strains. The plant-beneficial strain S6 carries a gene for a tRNA delta-isopentenylpyrophosphate (IPP) transferase domain which is not present in the strains S7 and S8. In *E. coli* this enzyme is involved in increasing spontaneous mutation frequency when cells need to adapt to environmental stress (Connolly and Winkler, 1989). Moreover, tRNA modifications mediated by tRNA delta-isopentenylpyrophosphate (IPP) transferase are required for virulence in *Shigella flexneri* by regulating posttranscriptional expression of the regulatory gene *virF* (Durand et al., 1997). The collagen-binding domain of bacterial collagenases is missing in the beneficial *P. ananatis* S6, although present in S7 and S8. This domain is a major component of the extracellular matrix (ECM) and plays a role in cell attachment, haemostasis, differentiation and bacterial adhesion in human and plant pathogens (Foster and Hook, 1998). Interestingly, in *Yersinia enterocolitica* it is a part of the pathogenic bacterial strategy for avoiding host response (Nummelin et al., 2004). The GMP synthase domain exclusively found in the pathogenic *P. ananatis* strain S7 is known to play an important role in cell-to-cell signaling in regulation of virulence in the plant pathogen *Xanthomonas campestris* (Ryan et al., 2006a). This domain is also involved in aggregative behavior, adhesion, biofilm formation, and the virulence of animal and plant pathogens (Ryan et al., 2006b). The role of these EDLs in the interaction of the three maize seed strains *P. ananatis* S7, S8, and S9 with maize plants remains unclear and merits further investigation.

Overall, our study showed that groups of bacterial endophytes with highly related genotypes but different phenotypes in terms of effects on host plants may exist in the same ecological niche. It can be expected that seed endophytes colonize, at least to a certain extent, plants derived from these seeds. Consequently, both, potential plant pathogens and mutualistic endophytes, may be together transmitted to the developing plant.

To predict the phenotype of plant-microbe interactions from traits manifested on the genome of bacteria is an attractive idea and would very much facilitate efforts in selecting microbial inoculants for improved plant production in sustainable agriculture. However, given the high genomic similarity between strains showing distinct phenotypes in regard to their interaction with plants, we conclude that plant pathogenicity and mutualism in *P. ananatis* may be based on rather subtle differences, e.g., on the expression of genes leading to plant defense reactions. Plant-bacteria interactions, irrespectively of whether pathogenic or beneficial must be considered as a multi-dimensional system and the expression of pathogenic or beneficial effects might depend on a multitude of parameters such as the plant/bacterial physiology, environmental conditions and/or a very fine-tuned interaction between bacterial elicitors and plant response.

### Conflict of interest statement

The authors declare that the research was conducted in the absence of any commercial or financial relationships that could be construed as a potential conflict of interest.
