# Supplementary material for: The genomes of closely related Pantoea ananatis maize seed endophytes having different effects on the host plant differ in secretion system genes and mobile genetic elements
Source: Front Microbiol. 2015 May 12;6:440. doi: 10.3389/fmicb.2015.00440 (PMC4428218; doi:10.3389/fmicb.2015.00440)
Supplement: Supplementary file 1 [file Table1.DOCX]

**Supplementary Table 1. Proportion (%) of sequence similarity between seven *P. ananatis* strains.** Proportions are given for the strains in each row in relation to the comparator strains in the top row.

| **Species** | **Strains** | S6 | S7 | S8 | AJ13355 | LMG20103 | LMG5342 | PA13 |
| --- | --- | --- | --- | --- | --- | --- | --- | --- |
| *P. ananatis* | S6 | 100 | 99.3 | 99.29 | 99.27 | 99.29 | 99.26 | 99.26 |
| *P. ananatis* | S7 | 99.3 | 100 | 99.31 | 99.29 | 99.34 | 99.28 | 99.3 |
| *P. ananatis* | S8 | 99.29 | 99.31 | 100 | 99.35 | 99.35 | 99.28 | 99.25 |
| *P. ananatis* | AJ13355 | 99.27% | 99.29% | 99.35 | 100 | 99.33 | 99.23 | 99.26 |
| *P. ananatis* | LMG20103 | 99.29 | 99.34 | 99.35 | 99.33 | 100 | 99.31 | 99.25 |
| *P. ananatis* | LMG5342 | 99.26 | 99.28 | 99.28 | 99.23 | 99.31 | 100 | 99.21 |
| *P. ananatis* | PA13 | 99.26 | 99.3 | 99.25 | 99.26 | 99.25 | 99.21 | 100 |
