# Supplementary figures and images for: The genomes of closely related Pantoea ananatis maize seed endophytes having different effects on the host plant differ in secretion system genes and mobile genetic elements
Source: Front Microbiol. 2015 May 12;6:440. doi: 10.3389/fmicb.2015.00440 (PMC4428218; doi:10.3389/fmicb.2015.00440)

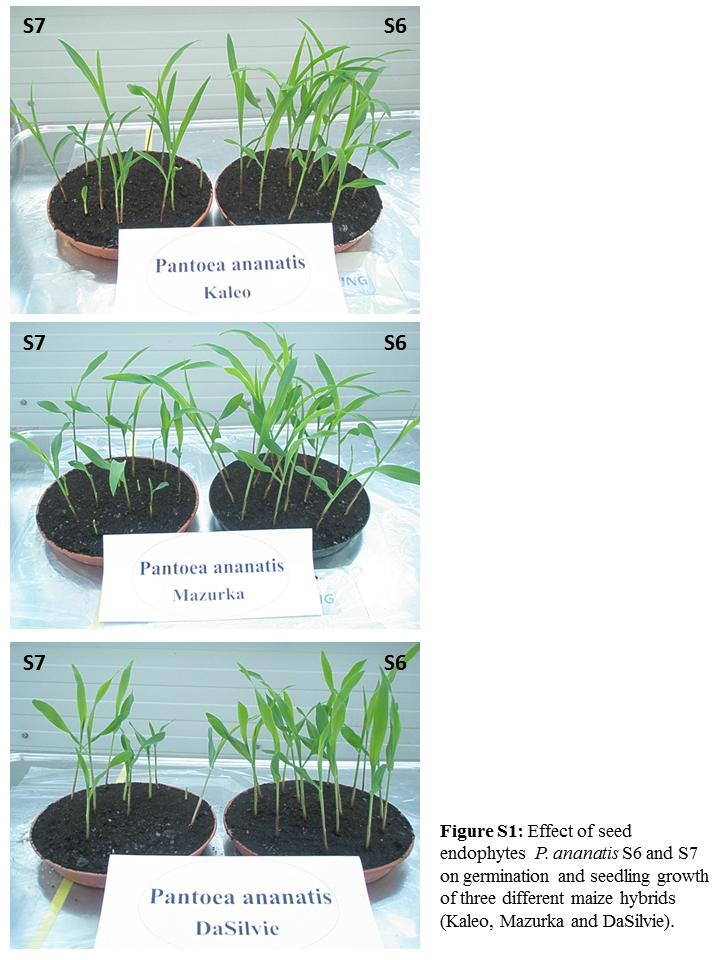

Supplement: Supplementary file 8 [file Image1.TIF]

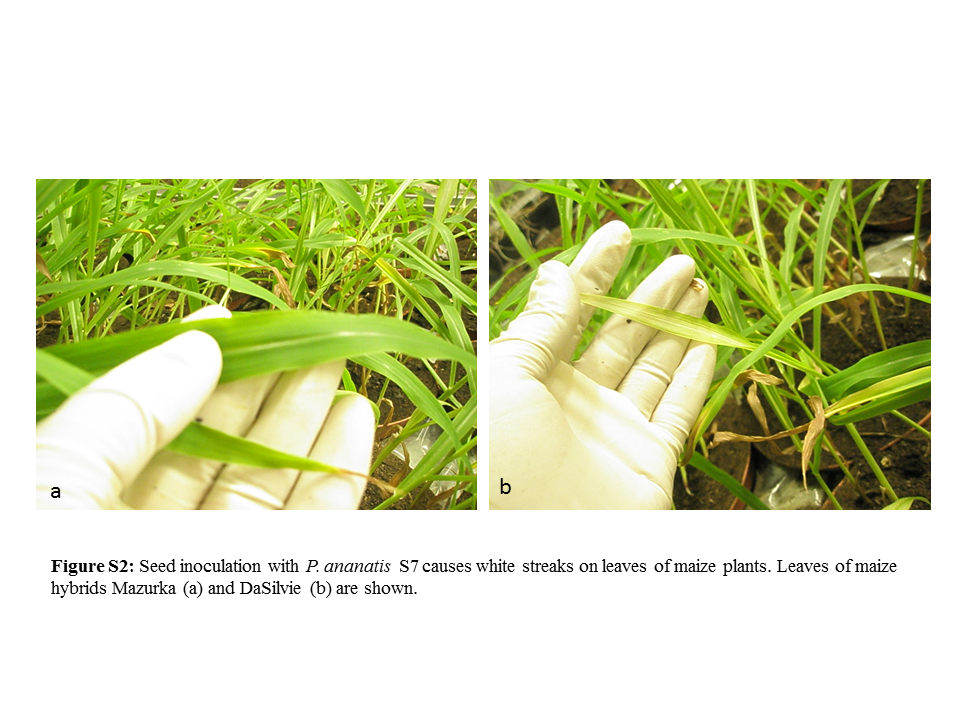

Supplement: Supplementary file 9 [file Image2.TIF]
